# The Pitfalls of Ascribing Moral Agency to Corporations: Public Obligation and Political and Social Contexts in the Commercial Determinants of Health

**DOI:** 10.1111/1468-0009.12678

**Published:** 2023-10-25

**Authors:** EDUARDO J. GÓMEZ, NASON MAANI, SANDRO GALEA

**Affiliations:** ^1^ Lehigh University; ^2^ University of Edinburgh; ^3^ Boston University

**Keywords:** commercial determinants of health, morals, policy interference, civil society, health policy

## Abstract

**Policy Points**

- Government and civil society should be held more accountable for creating food and beverage regulatory policies rather than assigning moral agency to the food and beverage industry.
- Nutrition policymaking institutions should ensure civil society's ability to design regulatory policy.
- Government policymaking institutions should be isolated from industry interference.

The rise of noncommunicable diseases (ncds) is a manifestation of a global economic system that currently prioritizes wealth creation over health creation. Many key problems and solutions lie outside the health sector. It is well established that commercial actors contribute to poor health through, for example, the production and marketing of harmful products such as calorie‐dense, nutrient‐poor foods. Some also believe that industries have been predatory in nature (i.e., marketing their products to vulnerable populations, such as children and the poor).^1^, ^2^ Commercial actors, through their industry representatives, have also been perceived as indirectly contributing to our poor health by seeking to shape the science (e.g., cherry‐picking and revealing data that question the relationship between sugar consumption and ill health) used to question the need for soda taxes^3^; the regulatory environments, such as lobbying against marketing restrictions or soda taxes or working with the government to cosponsor health campaigns and/or engage in product self‐regulation in turn generate government incentives and precedents not to pursue regulations.^4^, ^5^, ^6^ Commercial actors have also defended social norms, “weaponizing” issues such as defending individual liberties in the right to consume whichever products individuals desire in order to avoid regulations—deemed as “nanny state” intrusions—and to maximize future revenue.^7^

Although these industries activities are harmful, we must also acknowledge that industries may at times engage in illegal activities and that they should be held accountable for them, such as through the imposition of fines or public shaming. Recently, for example, for the first time, the US Food and Drug Administration (FDA) has filed complaints and a civil monetary penalty against e‐cigarette companies that ignored FDA warnings and did not receive authorization to sell their products.^8^ Industries therefore should realize that they have an obligation to obey the law and that governments can hold them accountable for their illegal actions and seek prosecution.

These kinds of activities have also led to a public perception that some major industries are malevolent or morally corrupt actors that intentionally strive to seek profit maximization at any cost, cutting legal corners and misleading the public to achieve their goals. Indeed, there is growing evidence for harmful product industries in particular that such strategies and tactics are widespread. In this essay, we focus on those industries that produce products that we consume, such as ultra‐processed foods, soda, and alcohol, that are associated with health ailments and risk factors, such as obesity.

Should we be viewing an industry as uniquely malevolent? Should we expect industrial actors to focus on the public's health at the expense of their profits? Particularly for large, publicly owned, profit‐seeking entities, there is a temptation to ascribe morality or will in the same way as we might the activities of an individual. This is understandable because corporations benefit from the notion of personhood in many ways and themselves seek to ascribe agency and morality in positive ways in their activities, particularly related to corporate social responsibility. Yet, at the core, all commercial activities, from supply chain management to employee benefits to corporate social responsibility, are driven by the same basic motive: profit. If this is “business as usual” for corporations, should they be held morally accountable for their actions?

Although we can establish causal pathways for harm arising from business practices, an alternative argument can be made that these industries and their powerful practices exist because government and society have afforded industries with such power. In this sense, government and society must also hold themselves accountable and refrain from assigning the moral agency to industry to “self‐regulate.” Indeed, presidents, congressional leaders, and citizens are the ones who must ultimately address a system of regulatory controls and incentives that contributes to industry's influence and social ills. Government and civil society create the policy rules and incentive structures that ultimately facilitate industry's political, policy, and social influence. For too long, many such rules have been framed as a given, an immovable baseline. From this perspective, government and civil society are also to blame for the commercial sector's harmful effects on public health. Therefore, the problem with assigning moral responsibility and agency to industry is that it absolves government and society of this responsibility. In this sense, we cannot falsely attach morality to blind for‐profit activity that is incentivized through broader political and social structures.

Seen in this light, researchers should instead consider taking a step back to first understand the political and social contexts that shape the government's willingness to establish policy incentive structures within which commercial industries operate. For example, what political incentives and/or barriers do presidents and congressional leaders have as they consider establishing laws and regulations that shape industrial activities and in turn protect society from the commercial sector's influence on our health? What incentives and opportunities do activists and academic researchers have to work among themselves and with the government to achieve this objective?

It seems that what this approach calls for is an *antecedent political and social approach* to establishing the policy incentive structures that commercial industries operate within. In this approach, government representatives and civil society have the power to enact laws and regulations that circumvent harmful industry ambitions, tactics, and harm. Although we certainly acknowledge the ongoing power and harm that industries have on population health, industry can only respond to the incentive structures that they are given by government and society.

In this article, we discuss the formation of the antecedent political and social conditions for government to establish these corporate incentive structures. We introduce an analytical approach that, acknowledging the core motivators of industry actors, seeks first and foremost to instead assign moral responsibility and agency with government and society and protect that responsibility from commercial influence. When viewed from this perspective, we believe that a corporation should be treated akin to an organism that responds to its environment. This suggests that we need to treat corporations as such and a) move away from ascribing to it moral values and responsibilities, and, instead, b) move toward constraining their broader social, political, and legal environments in ways that force them to live in equilibrium with it. It is in doing both that we can take moral, political, and social responsibility as governments and societies to then shape a better world. In making our claim, we draw from several case study examples and conclude this article with alternative political and policy recommendations.

## Moral Pitfalls and the Political and Social Origins of the Commercial Determinants of Health

For several years, scholars have been describing the role of the commercial sector in shaping public health policy. Known as the corporate political activity literature, this body of literature has explored how and to what extent commercial industries have become more involved in the policymaking process.^9^ More recently, researchers have underscored the various political and social tactics that these industries adopt to influence this process, such as lobbying, financial incentives, and building third‐party constituents, while sharing and working with policymakers.^10^, ^11^ Furthermore, there appears to be an underlying assumption, although not explicitly stated in these works, that these industries are behaving in an immoral manner: that is, intentionally placing their interests in profit maximization over the health of the population.

Some have recently recognized the immoral behaviors of these industries. Tempels and colleagues,^12^ for example, highlights the irresponsibility associated with the marketing and sales of some harmful products, with earlier work highlighting the immorality of industry marketing toward children and lobbying, though cautioning that we should also consider the positive steps that industries take to improve population health, such as improving the quality of foods sold.^13^ Indeed, for Tempels and colleagues,^12^ it is important that we do not overlook the positive role that industries contribute to health through their corporate social responsibility activities. Furthermore, Tempels and colleagues^12^ claim that industries must respect the consumer's individual autonomy, refrain from malevolent intentional or unintentional behavior, and reflect on broader morality issues rather than simply adhering to regulatory policy. Industries should also share social responsibility for safeguarding population health, going well beyond their social responsibility tactics.^12^, ^13^

The challenge with this literature, however, is that it falsely assigns moral responsibility to the commercial sector. How are we to assume that industries will not be self‐interested and behave in ways that we outside of industry perceive as immoral? How can we not assume that industries will strive to pursue a host of market and political strategies that favor their survival and success? This is especially the case in a context in which industries face an extensive amount of market competition and their ability to survive rests on their ability to find several market strategies that sustain their dominance.^14^

By way of example, consider the alcohol industry. The industry itself has been described as irresponsible in its approach to marketing to children and young adults and in its approach to combating evidence‐based policy. At the same time, the industry ascribes itself positive moral agency, proposing that it self‐regulate to ensure “responsible” marketing,^15^ and engaging in a range of corporate social responsibility initiatives, which themselves have been criticized as ineffective^16^, ^17^, ^18^, ^19^ and vectors of misinformation.^20^, ^21^, ^22^, ^23^ At the core, however, the industry as a whole is reliant on heavy alcohol consumers,^24^ and heavy drinking occasions,^25^ for the large majority of its sales. This fundamental conflict of interest (COI) and the tactics and strategies that emanate from it, are not a question of corporate responsibility, or irresponsibility. They are the result of a fundamental COI. If this is what “just business” looks like, then surely it is more important that we as a society isolate ourselves from the influence of that industry and reconsider what “just business” can or should be in this context rather than ascribe moral agency (positive or negative) to an industry that will continue inexorably to seek profit at the expense of health.

Therefore, in this article, we suggest an alternative set of questions and approach to addressing the commercial determinants of health. Our contention is that moral responsibility and agency should instead first reside with government and society as actors that can establish the interests, strategies, and, ultimately, the power needed to institute the policy environment within which industries operate. Put simply, government and society are the actors that should be able to create the constraining regulatory environment within which industries operate. It is therefore government and society that should determine to what extent industries shape our macrosocial environment, consumer preferences, and, ultimately, our health.

To better understand our perspective, we introduce an analytical framework titled an antecedent political and social approach to the commercial determinants of health. Grounded in political and social science theory, this framework establishes several antecedent conditions leading to the political and social conditions that ultimately create the regulatory laws and policies within which commercial industries operate.

### Establishing an Antecedent Political and Social Approach

Political scientists and sociologists have often approached state formation processes (i.e., the creation of laws and bureaucratic institutions) by explaining the preexisting political and social contexts and coalitions giving rise to their formation.^26^ In this approach, the formal design and presence of specific types of institutions, such as the bureaucracy and governing laws, do not bode well in predicting variation in policy outcomes and are not perceived as the primary independent variables. Instead, differences in historical political contexts, such as governing elite conflicts and the resulting political coalitions that emerge from them in turn shape the emergence of different types of institutions that often exhibit distinctive paths toward policy outcomes.^26^ Waldner's^26^ alternative analytical approach therefore focuses on examining the political and social origins of institutions and their effects on economic development.

In a similar approach, we advocate for an examination of the antecedent political and social contexts that shape government and civil societal interests and motivations to create a system of rules and regulations that in turn shape and constrain corporate industry incentive structures. This approach provides us with an opportunity to explain the origins of such government and civil societal interests and motivations, how they shape the formation of laws and regulatory institutions governing the commercial sector, and where moral agency should truly reside when it comes to the commercial determinants of health. Such an approach reveals that government and civil society are often more responsible for failing to enact the regulatory institutions needed to limit the commercial sector's harmful effects on our health (Table 1).

**Table 1:** Antecedent Political and Social Approach to CDoH

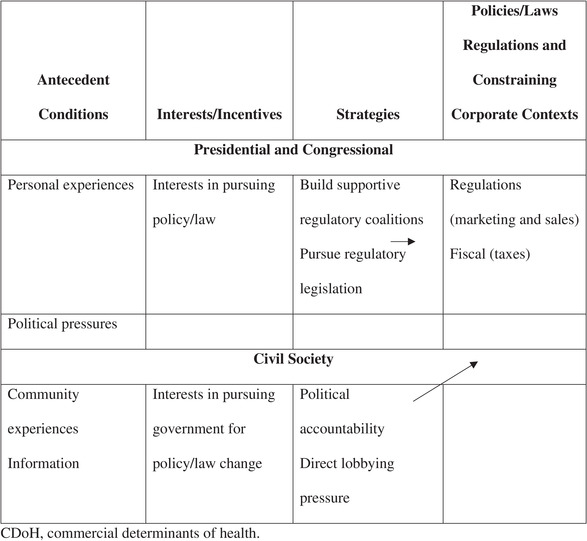

## Antecedent Political and Congressional Conditions

Historically, presidential interests and congressional coalitions have contributed to the formation of laws and institutions. In their seminal book *The Heart of Power: Health and Politics in the Oval Office*, Blumenthal and Morone^27^ explain that behind every piece of health care legislation in the United States lies the personal experiences, ideas, and interests of presidents. Throughout US history, presidential ideas have emerged from personal experiences, such as personal health struggles, to pressures from family members and friends—Franklin D. Roosevelt's struggle with polio stands out as a good example; moreover, these factors have motivated presidents to pursue their ideas amidst constraining institutional and economic contexts. Presidents also have the power to set the policy agenda: as Blumenthal and Morone^27^ explain, they can either go public, using the media to inform society and claim health policy credit, or they can strategically use the inner workings of the congress.

Decades later, one can see the importance of a president's personal experiences, interests, and policy incentives during the Presidents George W. Bush and Barack Obama administrations. Although President Bush's personal health experiences motivated him to pursue a healthy lifestyle and support physical fitness and good health, the Obamas’ personal experiences with their children's weight challenges also generated similar motivations.^28^ Indeed, First Lady Michelle Obama's concern about her daughter's weight and wellness in part motivated her to prioritize the creation of the national “Let's Move” initiative, a federal campaign that worked with local communities to address and reduce childhood obesity. Through this campaign, First Lady Obama's personal experiences and concerns ultimately led to building support for several initiatives; this included the creation of the first Task Force on Childhood obesity, federal programs that improved the quality of food within schools and access to school physical fitness initiatives.^29^ Over time, however, some criticized the First Lady and the White House for not speaking up about proposed federal agency food regulations after intensive industry lobbying efforts, a shift in Michelle's childhood obesity campaign to emphasize the importance of exercise in lieu of regulation and blaming the food and beverage industry, with some believing that she became too close to the industry.^30^ Although Michelle's political actions were admirable and a good example of the potential benefits of personal leadership interests in defense of children's health, more could have been done to defend and strengthen her “Let's Move” campaign, in turn highlighting the importance of ongoing political commitment.

These political factors are also important in other countries. Arguably one of the most successful cases of government food and beverage marketing and labeling regulations in the Americas has been the case of Chile. There, despite several years of intensive and successful industry opposition, eventually the government proved successful in adopting restrictions on children's advertising of unhealthy food products as well as more effective front‐of‐package food labels.^31^ Some have argued that it was the personal beliefs and interests of an influential senator, Guido Girardi (Party for Democracy), who was also president of the Senate's Health Commission, that motivated him to successfully build consensus for reform within the government.^32^, ^33^ Senator Girardi also enlisted a human rights discourse when arguing for the adoption of these policies, comporting with civil society's views, claiming that children had the right to be protected from junk food advertising. Studies have shown that it was the interests and actions of this influential policy entrepreneur, in addition to the presence of supportive government institutions, academics, and activists, that contributed to Chile's eventual success in adopting perhaps the most stringent regulatory actions against industry to date in the western hemisphere.^33^

Alternatively, political leaders’ personal experiences and views may not at times favor the creation of effective regulatory policies. Consider the case of Mexico. This country provides a vivid example of the importance of understanding presidential personal experiences, interests, and motivations. There, presidential interests and experiences have historically shaped the government's relationship with the soda industry and national NCD policy. For example, President Vincente Fox (National Action Party, 2000–2006) was previously a Coca‐Cola executive. Fox's relationship with the US soda industry appears to have shaped his ideas and interests in neglecting to pursue soda regulations, whereas his connections with the industry facilitated its access to policymakers.^5^ In a context in which the policymaking process has been highly influenced by the president and government officials, Fox's and other Mexican presidents’ personal experiences and ideas have mattered considerably.

Therefore, presidents and aligned policymakers may also behave in an immoral manner. Because of their personal connections with businesses and even their own investments in the food and beverage sector—as seen in South Africa^34^—this can motivate presidents to prioritize corporate profits and grow the economy, ultimately benefiting them while indirectly supporting those industries that are harmful to our health.^5^

Finally, it is important to emphasize that government and business actors are at times part of the same policy system.^35^ Although this may not be the case with respect to presidential and/or congressional efforts to impose taxes on beverages and foods, this is often the case with respect to government–industry partnerships to improve the nutritious content of foods^36^ and government‐authorized agreements for industry self‐regulation.^35^ Furthermore, federal regulatory agency allowance of industry representatives to sit on policy “working groups” when considering regulations can be perceived as suspect because of businesses’ influence over policy decisions.^5^ In both instances, the public may have less trust in government to devise policy autonomously and in the public's interests.

Going forward, health officials and activists can enlist the support of influential international organizations to question the appropriateness of these government–industry partnerships, which is often the source of COI. For example, the World Health Organization (WHO) is committed to helping governments prevent and manage COIs in nutrition policy, recently offering a draft *Decision‐Making Process and Tool* to help governments achieve this.^37^ Health officials and activists can harness the WHO's commitment, legitimacy, and influence to apply further pressure onto governments to adopt this *Tool* and to cease engaging in these COI activities.

Thus, in sum, better understanding the political antecedents has the potential to provide additional insight into why some governments pursue regulatory policies constraining industry's policy influence and success versus those that do not.

## Antecedent Social Conditions

What are the antecedent social conditions that shape the rise of civil society's interests, incentives, and strategies for pursuing policies and laws that establish the regulatory institutions within which industries operate? Addressing this question requires that we first address the origins of community motivations and interests.

As seen at the political level, community experiences can certainly shape community interests in working with government to pursue policies and laws. For example, increased community awareness about the growing prevalence of childhood obesity and its association with junk foods can prompt community efforts to address this issue. In the United States, Fleming‐Milici and Harris^38^ maintain that parents have become increasingly concerned about their children's exposure to television advertisements for unhealthy foods and that this concern in turn has prompted demands for government regulatory action. Relatedly, in poor urban areas, community awareness of the lack of sufficient access to quality foods and the barriers to obtaining such foods have been known to cause community stress.^39^ Like what is seen at the national political level, community interests in these and related commercial issues are often driven by the publication of data and public awareness campaigns.

When motivated by these interests and concerns, civil societal actors have pursued several strategies to approach government and pursue policy reform. When the political and institutional contexts permit, communities (such as through nongovernmental organizations and/or public health advocacy groups) can hold their governments accountable for the failure to introduce policies and regulations that increasingly constrain the commercial sector's ability to shape downstream consumer preferences and community health.

However, there are several ongoing challenges to civic mobilization and pressuring government for regulatory action. Chief among these challenges is having the resources needed to mobilize concerned advocacy groups and citizens while garnering a sufficiently large coalition of supporters. Yet another challenge is having reliable access to national or subnational bureaucratic and/or congressional institutions in which citizens can convey their interests, frustrations, and demands. As seen in Brazil, however, when local communities have access to these types of participatory institutions, health budgets and policies can be designed in an effective and equitable manner.

To what extent is civil society engaging and pressuring presidents and the congress for more aggressive regulatory action toward the commercial sector? Progress is certainly being made along these lines, with communities and activist organizations in the United States, Europe, and Latin America making considerable progress. Especially in resource‐poor settings, however, international organizations and philanthropic institutions could do more in providing support to these groups.

Finally, civil societal actors, such as university researchers, also need to ensure that they are refraining from accepting funding from corporations. In the area of gambling, a sector that has received significantly less attention in the commercial determinants of health, researchers have highlighted the problems with biased academic research because of corporate funding and/or tax revenues from gambling activity.^40^ Those funded from these sources often downplay the problem of gambling advertising and the need for regulations.

## Industry Power Revisited

Understanding the political and social antecedents to regulating the commercial sector suggests an alternative perspective with respect to power and the commercial determinates of health. When the president and the government commit to pursuing industry regulations, reinforced with pressures from civil society, this establishes the regulatory constraints within which industries must operate. For example, successful efforts to impose marketing and advertising regulations for unhealthy food products limit industries’ choices and behaviors in the market. Industries can only strive to maximize their profits in a context of regulatory policies that government and society establish. When viewed from this perspective, power in the commercial determinants of health rests with government and civil society, not necessarily with the commercial sector.

However, it is only through a historical analysis of the political and social antecedents to policymaking processes that we can understand this alternative perspective. The challenge with the existing corporate political activity and commercial determinants of health literature is that it often takes a historical approach to research, focusing on the contemporary activities of industries and their efforts to undermine policy, markets, and society.^41^ Moreover, this literature neglects the a priori political and social drivers determining the industry's capacity to maneuver and have influence. Our work here suggests that power, in addition to moral responsibility, may instead reside with government and civil society.

## Conclusion

In closing, several key lessons emerge from this study. First, we must be careful not to immediately assign moral responsibility to the commercial sector. There is often an underlying assumption that certain industries are immoral actors seeking to advance their profits over and above population health concerns. Although we do not disagree with the assumption that industries prioritize profit over health, scholars should also recognize that it may be misleading to immediately blame industry, as it distracts from the underlying incentives that govern their behavior. Perhaps we should instead assign moral responsibility to the makers of policy regulations and the incentive structures within which industries operate (i.e., government and society). Ultimately, it is our presidents and congressional leaders who are primarily responsible for creating the regulatory environment within which industries function.

Nevertheless, it is also important to recognize that, at times, government and civil societal actors may act immorally; when compared with the commercial sector, however, far less attention has been paid to this issue. In the commercial determinants of health, there are arguably fewer documented instances in which government and society reach out to businesses for personal gain at the expense of public health. Furthermore, unlike businesses, government and society are not always expected to act immorally with respect to seeking their self‐interests—it's not “business as usual” for them. Indeed, there are many if not more government and civil societal actors avoiding immoral policy behavior who are committed to improving our health when compared with the commercial sector. Thus, our criticism of wrongfully assigning moral blame to the commercial sector should not be done for government and society, which in turn further justifies our need to apply a moral lens to these actors.

Additionally, we have learned that it is the government and civil society that gives industry the power to operate and influence policy and population health. This power emanates from the political and social willingness to create effective regulatory policies that constrain industry's behaviors and opportunities for action. Therefore, in contrast to other studies,^42^, ^43^ our alternative perspective suggests that power should emanate from government and civil society, which in turn can shape the policy environment within which industries operate. As Nestle^44^ claims, coalitions, often created by society, can muster the ability to pursue regulatory policies that can potentially limit industry's harmful effects on our health. As Nestle^44^ further claims, “Even seemingly weak advocacy groups can harness their power to effect change when they share a compelling vision, organize community support, and build coalitions.”

Finally, civil society also has the power to impress onto commercial enterprises the importance of behaving in a morally responsible manner: giving back to society, which is at the heart of civic demands for corporate social responsibility. Society has a long history of expecting corporations to behave in this manner, shaped by unique political, social, and environmental contexts.^45^ Given that businesses at times have acted in an altruistic manner, such as Unilever's efforts in the 1990s to improve the nutritious quality of their products by removing trans fats from margarines and spreads without an immediate market incentive to do so,^13^ it seems that businesses do have the capacity for moral agency. During these periods, society can help in holding corporate boards accountable for behaving in an ethical and responsible manner.

Going forward, we need to focus more on the political and social failures to transform and improve the regulatory policy context within which industries operate. For example, why is there no ongoing political will to impose effective marketing and sales regulations and even a national soda and junk food tax in some countries, such as the United States? To address this question, we need to take a step back and look at the personal experiences, incentives, and motivations of our political leaders; moreover, we need to step back and look at the willingness and ability of civil society at large to pressure their leaders into taking these much‐needed policy actions.

*Conflict of Interest Disclosures*: No disclosures were reported.
